# The effect of adjuvant chemoradiotherapy on survival after R0 resection for stage III-N2 nonsmall cell lung cancer: A meta-analysis

**DOI:** 10.1097/MD.0000000000029580

**Published:** 2022-07-15

**Authors:** Dailong Li, Wanqiang Li, Yaqi Pang, Lu Xu, Xinhua Xu

**Affiliations:** a Department of Oncology, Yichang Central People’s Hospital, The First College of Clinical Medical Science, China Three Gorges University, Yichang, China; b Department of Urology, The First College of Clinical Medical Science, China Three Gorges University, Yichang Central People’s Hospital, Yichang, China; c Department of Radiation Oncology and Medical Oncology, Zhongnan Hospital of Wuhan University, Wuhan, China.

**Keywords:** meta-analysis, nonsmall cell lung cancer, N2-stage, adjuvant postoperative chemoradiotherapy

## Abstract

**Background:**

Adjuvant chemotherapy is still the standard treatment for stage III-N2 nonsmall cell lung cancer after R0 resection, and it is still controversial whether conventional adjuvant radiotherapy is needed. We used meta-analysis to try to answer whether adjuvant postoperative chemoradiotherapy (POCRT) can bring survival benefits to patients with stage III-N2 nonsmall cell lung cancer after R0 resection.

**Methods:**

Up to June 25, 2021, the databases of PubMed, Embase, Cochrane Library, CNKI, and Wanfang were searched, and clinical studies on POCRT for stage III-N2 nonsmall cell lung cancer were included. RevMan5.4 software was used for meta-analysis.

**Results:**

A total of 8959 patients were included in 5 randomized controlled trials and 17 retrospective studies. The results of the meta-analysis showed that POCRT could improve 3 and 5 years overall survival (OS) rate (OR = 1.52, 95%CI: 1.05–2.20; OR = 1.30, 95%CI: 1.16–1.46), 3 and 5 years disease-free survival (DFS) rate (OR = 1.34, 95%CI: 1.01–1.76; OR = 1.74, 95%CI: 1.43–2.12), and 5-year locoregional recurrence-free survival (LRFS) rate (OR = 2.69, 95%CI: 1.76–4.11) in patients with stage III-N2 nonsmall cell lung cancer compared with adjuvant postoperative chemotherapy (POCT) alone. But could not improve 5-year distant metastasis-free survival (DMFS) rate (OR = 1.14, 95%CI: 0.52–2.52). The results of subgroup analysis showed that postoperative sequential chemoradiotherapy could improve the 3 and 5 years OS rate (OR = 2.06, 95%CI: 1.22–3.46; OR = 1.39, 95%CI: 1.21–1.59). Three-dimensional conformal radiotherapy (3DCRT) or intensity-modulated radiotherapy (IMRT) can improve the 3 and 5 years OS rate (OR = 1.80, 95%CI: 1.09–2.99; OR = 1.31, 95%CI: 1.04–1.66). In addition, POCRT could improve the 3-year OS rate (OR = 1.88, 95%CI: 1.21–2.92) in patients with N2 single-station lymph node metastasis compared with POCT alone.

**Conclusion:**

Compared with POCT alone, adjuvant POCRT can significantly improve the overall survival rate of patients with NSCLC after R0 resection of stage III-N2, especially in patients with N2 single-station lymph node metastasis. Accurate radiotherapy techniques such as 3DCRT or IMRT are recommended, and postoperative sequential chemoradiotherapy is the best treatment mode.

## 1. Introduction

Nonsmall cell lung cancer (NSCLC) accounts for 80% of the total lung cancer, of which stage III-N2 lung cancer accounts for about 30% of the total NSCLC.^[1,2]^ Stage III-N2 NSCLC is a highly heterogeneous disease, and the prognosis of the patients varies greatly.^[3]^ The prognosis of patients with complete resection of tumor (R0) was better than that of patients without complete resection of tumor (R1 and R2). However, even in patients with stage III-N2 NSCLC who underwent R0 resection, the 5-year overall survival (OS) rate was only 6–35%, and the rate of distant metastasis was more than 50%.^[4,5]^ High-level clinical evidence shows that postoperative chemotherapy (POCT) can significantly prolong survival, which is the standard treatment for stage N2 NSCLC.^[6,7]^ However, some studies have shown that even with R0 resection and active adjuvant POCT, the local recurrence rate is still as high as 20–40%.^[8,9]^ As a local treatment, postoperative radiotherapy can effectively kill potential subclinical lesions, improve local control rate, and further improve the long-term survival of patients.^[10,11]^ Unfortunately, the heart, lung and other important organ injuries caused by radiotherapy will affect the survival benefits of early NSCLC patients. High-level evidence-based medicine evidence confirmed that stage Ⅰ and Ⅱ NSCLC did not need adjuvant postoperative radiotherapy after R0 resection, but it is still controversial whether postoperative radiotherapy can bring survival benefits to patients with stage III-N2 NSCLC after R0 resection.^[12]^ With the application of new radiotherapy techniques, such as 3-dimensional conformal radiotherapy (3DCRT) and intensity-modulated radiotherapy (IMRT), not only the tumor target can be accurately irradiated, but also the surrounding normal tissues and organs can be protected to the maximum.^[13]^ The value of postoperative radiotherapy for patients with stage N2 NSCLC is being reconsidered by clinicians. Therefore, we use meta-analysis to try to answer the adjuvant chemotherapy combined with radiotherapy after R0 resection, that is, adjuvant postoperative chemoradiotherapy (POCRT), whether it can bring survival benefits to patients with stage III-N2 NSCLC.

## 2. Materials and Methods

### 2.1. Publication search

This meta-analysis was performed according to the Preferred Reporting Items for Systematic Reviews and Meta-Analyses (PRISMA) statement. The systematic literature search was performed through PubMed, EMBASE, Cochrane Library, CNKI, and WanFang database, covering all articles published up to June 25, 2021. The following keywords were used to retrieve articles: nonsmall cell lung cancer, NSCLC, postoperative, and radiotherapy. References of the retrieved publications were also screened. Only published studies with full-text articles were included. The search strategy for PubMed is described as follows:

#1 “Carcinoma, Non-Small-Cell Lung” [Mesh]

#2 “Nonsmall Cell Lung Cancer” OR “Non-Small Cell Lung Cancer” OR “Non-Small Cell Lung Carcinoma” OR “Carcinoma, Non-Small Cell Lung” OR “Non Small Cell Lung Carcinoma” OR “Non-Small-Cell Lung Carcinoma” OR “Non-Small-Cell Lung Carcinomas” OR “Lung Carcinomas, Non-Small-Cell” OR “Lung Carcinoma, Non-Small-Cell” OR “Carcinomas, Non-Small-Cell Lung” OR “Carcinoma, Non Small Cell Lung” [Title/Abstract]

#3 #1 OR #2

#4 “postoperative” OR “after operation” [Title/Abstract]

#5 “radiotherapy” OR “radiation therapy” OR “radiotherapeutics” [Title/Abstract]

#6 #3 AND #4 AND #5

The Cochrane Library, EMBASE, CNKI, and Wanfang database used similar search formulae.

### 2.2. Literature inclusion and exclusion criteria

#### 2.2.1. Inclusion criteria.

(1) Clinical study, divided into adjuvant POCRT group and POCT group alone; (2) patients with pathologically diagnosed stage III-N2 NSCLC, underwent R0 resection; (3) there are extractable outcome indicators, such as OS, DFS, LRFS, and DMFS, etc; (4) the literature is the original research and can provide the original data.

#### 2.2.2. Exclusion criteria.

(1) reviews, case reports, conference summaries, nonclinical reports and repeated studies; (2) the data are incomplete and the original data are not available. (3) the number of cases <30; (4) Low-quality literatures with Jadad scores <3 or NOS scores <6.

### 2.3. Data extraction and literature quality evaluation

The 2 investigators sift through the literature, extract the data, and cross-check independently to ensure that the data extracted from the literature are consistent. These literatures are screened strictly according to the inclusion and exclusion criteria. If there is any disagreement, it is decided by discussing or referring to the opinion of the third researcher. The randomized controlled trial was scored by Jadad scale,^[14]^ and the retrospective study was scored by NOS scale.^[15]^

### 2.4. Statistical analysis

The Review Manager version 5.4 software was applied to analyze the data. Results were showed with odds ratios (OR) and 95% confidence intervals (95%CI) and Meta-analysis forest map was drawn. Fixed-effects model was adopted when there was no evidence of significant heterogeneity (*P* > .1 and I^2^ < 50%); otherwise, random-effects model was used. Sensitivity analysis was used to test the stability of the results and funnel plots were used to evaluate publication bias. If possible, heterogeneity was explored and subgroup analyses were performed. All *P* values were 2-sided, and *P* < .05 was considered significant.

## 3. Results

### 3.1. Literature search and study characteristics

**Figure 1.**
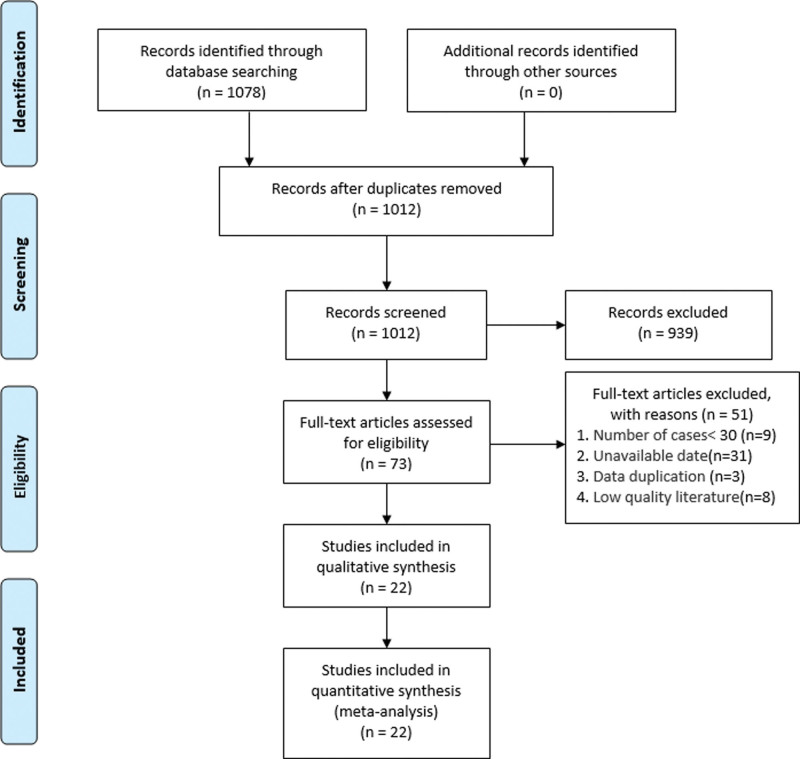
Literature screening flow chart.

A total of 1078 articles were retrieved, and 66 repeated articles were excluded by title, year, and author information. then after reading abstracts and full-text screening, 990 articles that did not meet the criteria were excluded and finally included 22 studies^[16–37]^ (Fig. 1). There were 8959 patients with stage III-N2 NSCLC, of which 3356 patients received POCRT and 5603 patients received POCT alone. The quality evaluation of the included studies is shown in Tables 1 and 2, all of which are of high quality. The key baseline characteristics of patients are fully described in all the included studies, as shown in Table 3.

**Table 1:** The included randomized controlled trials were scored according to the Jadad scale.

Author	Published date	Randomization	Blind	Withdrawal and loss of follow-up	Total score
Perry	2007	2	1	0	3
WY Shen	2014	2	1	0	3
JT Wu	2009	2	1	1	4
ZG Hui	2021	2	2	1	5
JM Sun	2017	2	1	1	4

**Table 2:** The included retrospective studies were scored according to the NOS scale.

										Study								
Category	Entries	①	②	③	④	⑤	⑥	⑦	⑧	⑨	⑩	⑪	⑫	⑬	⑭	⑮	⑯	⑰
	Representation of the exposure cohort	☆	☆	☆	☆	☆	☆	☆	☆	☆	☆	☆	☆	☆	☆	☆	☆	☆
Section	Representation of the nonexposed cohort	☆	☆	☆	☆	☆	☆	☆	☆	☆	☆	☆	☆	☆	☆	☆	☆	☆
	Determination of exposure																	
	No outcome event occurred before the study began	☆	☆	☆	☆	☆	☆	☆	☆	☆	☆	☆	☆	☆	☆	☆	☆	☆
Comparability	Comparability of cases and controls on the basis of the design and analysis	☆	☆	☆	☆	☆	☆	☆	☆	☆	☆	☆	☆	☆	☆	☆	☆	☆
	Results determination method	☆	☆	☆	☆	☆	☆	☆	☆	☆	☆	☆	☆	☆	☆	☆	☆	☆
Outcome	Adequate follow-up time	☆	☆		☆	☆		☆	☆	☆	☆	☆					☆	
	Complete follow-up	☆	☆	☆	☆	☆	☆	☆	☆	☆	☆	☆	☆	☆	☆	☆	☆	☆
Total scores		7	7	6	7	7	6	7	7	7	7	7	6	6	6	6	7	6

Notes: ① Wei W; ② HH Dai; ③ Kim; ④ LY Su; ⑤ Y Liu; ⑥ CY Mao; ⑦ L Deng; ⑧ X Zhang; ⑨ YL Li; ⑩ W Han; ⑪ Zou; ⑫ Wen Feng; ⑬ Fei Gao; ⑭ J Jin; ⑮ Douillard; ⑯ Alex Herskovic; ⑰ Hyojung Park.

**Table 3:** Basic characteristics of the included literature.

Study	Number of patients	Region	Research design	POCRT /POCT	Median radiation dose	Radiotherapy technique	Median follow-up time (mo)	Stage	Main outcome indicators
W Wei 2020	183	China	Retrospective	78/105	50Gy	IMRT	38	III-N2	②⑤
HH Dai 2011	221	China	Retrospective	96/125	NA	3DCRT and 2DRT	35.1	IIIA-N2	①②③④⑤⑥
Kim 2014	219	Korea	Retrospective	41/178	54Gy	3DCRT and 2DRT	NA	III-N2	②④
LY Su 2019	175	China	Retrospective	60/115	NA	3DCRT and IMRT	48	III-N2	①②⑦
Y Liu 2013	126	China	Retrospective	70/56	52Gy	3DCRT and IMRT	27.8	IIIA-N2	①②
JT Wu 2009	48	China	RCT	24/24	NA	3DCRT	NA	IIIA-N2	①③
CY Mao 2010	100	China	Retrospective	40/60	NA	3DCRT and 2DRT	NA	IIIA-N2	①②
X Zhang 2010	52	China	Retrospective	29/21	NA	3DCRT	28.5	III-N2	①
YL Li 2018	97	China	Retrospective	33/64	55Gy	3DCRT	29	IIIA-N2	②⑧
W Han 2017	313	China	Retrospective	23/90	50Gy	3DCRT and IMRT	38.8	IIIA-N2	①②
Zou 2010	183	China	Retrospective	104/79	50Gy	3DCRT and 2DRT and IMRT	72	III-N2	②④⑤
L Deng 2018	804	China	Retrospective	276/528	50 Gy	3DCRT and IMRT	32.07	IIIA-N2	②④⑤⑥
Perry 2007	37	America	RCT	19/18	NA	NA	41.5	IIIA-N2	①
WY Shen 2014	137	China	RCT	66/69	NA	3DCRT and IMRT	45	IIIA-N2	②④
W Feng 2015	357	China	Retrospective	70/287	50.4Gy	3DCRT	NA	IIIA-N2	①②③④⑥
F Gao 2020	2479	China	Retrospective	1290/1189	NA	NA	NA	IIIA-N2	②
M. Chen 2020	440	China	Retrospective	223/217	50.0Gy	3DCRT and IMRT	NA	III-N2	⑦⑧
Douillard 2008	118	America	Retrospective	48/70	NA	NA	NA	III-N2	②
ZG Hui 2021	310	China	RCT	140/170	50.0Gy	3DCRT and IMRT	NA	IIIA-N2	①②③④
JM Sun 2017	101	Korea	RCT	52/50	50.0Gy	3DCRT	NA	III-N2	①②③④
Alex Herskovic 2017	2691	America	Retrospective	516/2175	NA	NA	32.23	IIIA-N2	①②
Hyojung Park 2016	135	Korea	Retrospective	64/71	50.0Gy	3DCRT	NA	III-N2	①②③④⑤⑥

Notes: 2DRT = two-dimensional general radiotherapy, 3DCRT = three-dimensional conformal radiotherapy, IMRT = intensity modulated radiotherapy, ① 3-year overall survival, ② 5-year overall survival, ③ 3-year Disease-free survival, ④ 5-year Disease-free survival, ⑤ 5-year locoregional recurrence-free survival, ⑥ 5-year distant metastasis-free survival, ⑦ 3-year overall survival (N2 single station lymph node metastasis), ⑧ 3-year overall survival (N2 multiple stations lymph node metastasis).

### 3.2. OS

**Figure 2.**
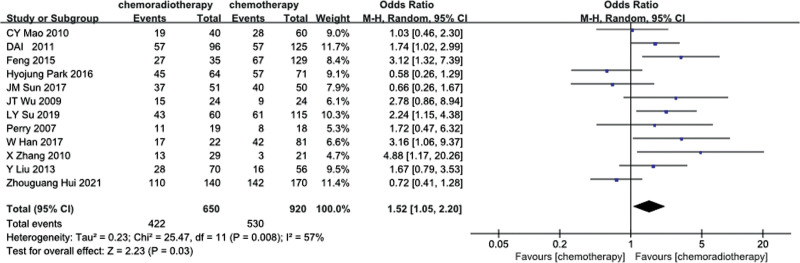
Three-year OS rate of the POCRT group vs the POCT alone group.

**Figure 3.**
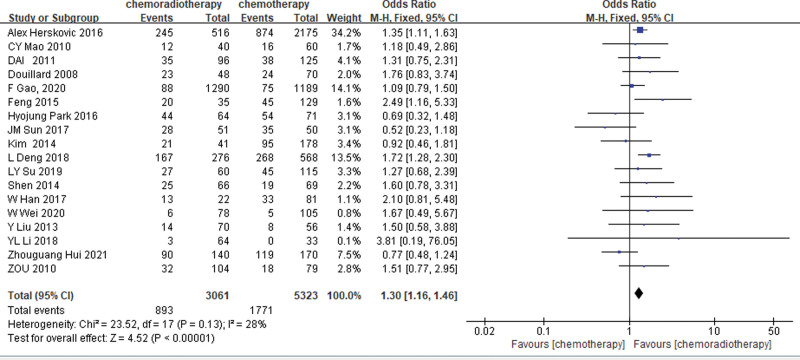
Five-year OS rate of the POCRT group vs the POCT alone group.

In total, 12 studies^[16,17,19,20,22,24,28,30,32,34,35,37]^ and 18 studies^[16,18,20–23,25–30,32–37]^ provided 3 and 5 years of OS data, respectively. Based on the results of the heterogeneity test (3-years OS: *P* = .008, I^2^ = 57%; 5-years OS: *P* = .13, I^2^ = 28%), 3-year OS rate was analyzed by random-effects model, and 5-year OS rate was analyzed by fixed-effects model. Meta-analysis showed that in stage III-N2 NSCLC, the 3-year and 5-year OS rate of the POCRT group was higher than that of the POCT alone group (OR = 1.52, 95%CI: 1.05–2.20, *P* = .03; OR = 1.30, 95%CI: 1.16–1.46, *P* < .00001). As shown in Figures 2 and 3.

### 3.3. DFS

**Figure 4.**
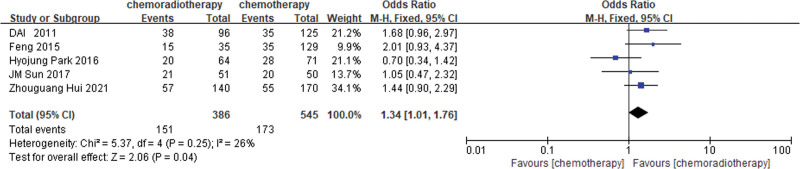
Three-year DFS rate of the POCRT group vs the POCT alone group.

**Figure 5.**
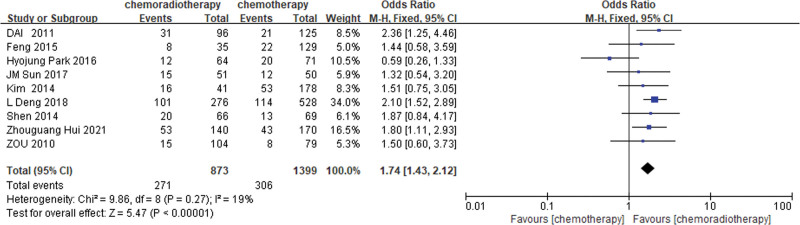
Five-year DFS rate of the POCRT group vs the POCT alone group.

An estimated 5 studies^[22,30,34,35,37]^ and 9 studies^[21–23,25,26,30,34,35,37]^ provided 3 and 5 years of DFS data, respectively. Based on the results of the heterogeneity test (3-year DFS: *P* = .26, I^2^ = 25%; 5-year DFS: *P* = .27, I^2^ = 19%), the results of the fixed-effects model analysis showed that the 3 and 5 years DFS rate of the POCRT group was higher than that of the POCT alone group (OR = 1.34, 95%CI: 1.01–1.76, *P* = .04; OR = 1.74, 95%CI: 1.43–2.12, *P* < .00001) (Figures 4 and 5).

### 3.4. LRFS

**Figure 6.**
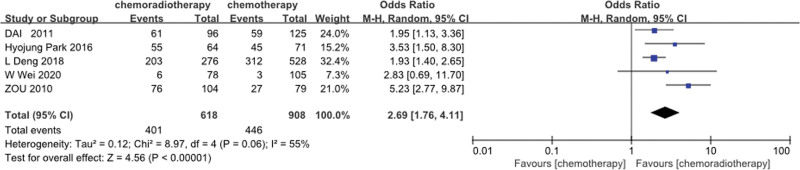
Five-year LRFS rate of the POCRT group vs the POCT alone group.

5 studies^[21,25,29,30,37]^ provided 5 years of LRFS data. Based on the results of the heterogeneity test (*P* = .06, I^2^ = 55%), the results of the random-effects model analysis showed that the 5-year LRFS rate of the POCRT group was higher than that of the POCT alone group (OR = 2.69, 95%CI: 1.76–4.11, *P* < .00001) (Fig. 6).

### 3.5. DMFS

**Figure 7.**
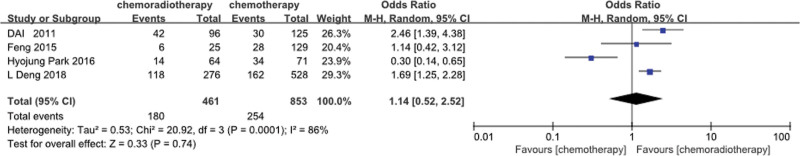
Five-year DMFS rate of the POCRT group vs the POCT alone group.

A total of 4 studies^[21,22,30,37]^ provided 5 years of DMFS data. Based on the results of the heterogeneity test (*P* = .0001, I^2^ = 86%), the results of random-effects model analysis showed that there was no significant difference in 5-year DMFS rate between the POCRT group and the POCT group (OR = 1.14, 95%CI: 0.52–2.52, *P* = .74) (Fig. 7).

### 3.6. Results of subgroup analysis of OS

To screen out the population most likely to benefit from adjuvant POCRT and the best treatment model, we performed a subgroup analysis of OS based on the combination mode of postoperative chemotherapy and radiotherapy, radiotherapy technology, and lymph node metastasis status at the N2 station. The results of subgroup analysis showed that postoperative sequential chemoradiotherapy could improve the OS rate at 3 and 5 years (OR = 2.06, 95%CI: 1.22–3.46, *P* = .007; OR = 1.39, 95%CI: 1.21–1.59, *P* < .00001), while postoperative concurrent chemoradiotherapy could not improve the OS rate at 3 and 5 years (OR = 1.17, 95%CI: 0.67–2.05, *P* = .58; OR = 1.20, 95%CI: 0.84–1.71, *P* = .33). 3DCRT and IMRT can improve OS rate at 3 and 5 years (OR = 1.80, 95%CI: 1.09–2.99, *P* = .02; OR = 1.31, 95%CI: 1.04–1.66, *P* = .02). In addition, compared with POCT, POCRT can significantly improve the 3-year OS rate of patients with N2 single-station lymph node metastasis (OR = 1.88, 95%CI: 1.21–2.92, *P* = .005), but has no obvious advantage for patients with N2 multi-station lymph node metastasis (OR = 1.64, 95%CI: 0.89–3.02, *P* = .11) (Tables 4 and 5).

**Table 4:** Three-year OS rate subgroup analysis.

	Number of studies	Heterogeneity	OR 95%CI	*P*
The combination mode of postoperative chemotherapy and radiotherapy				
Sequential chemoradiotherapy	7	*P* = 0.02, I^2^ = 60%	2.06 (1.22–3.46)	0.007
Concurrent chemoradiotherapy	3	*P* = .17, I^2^ = 43%	1.17 (0.67–2.05)	0.58
Others	2	*P* = .16, I^2^ = 48%	0.88 (0.31–2.47)	0.81
Radiotherapy technology				
3DCRT or (and) IMRT	8	*P* = .008, I^2^ = 63%	1.80 (1.09–2.99)	0.02
Mix	2	*P* = .29, I^2^ = 11%	1.47 (0.91–2.37)	0.12
Others	2	*P* = .16, I^2^ = 48%	0.88 (0.31–2.47)	0.81
N2 station lymph node metastasis				
Single-station lymph node metastasis	2	*P* = .28, I^2^ = 14%	1.88 (1.21–2.92)	0.005
Multi-station lymph node metastasis	2	*P* = .42, I^2^ = 0%	1.64 (0.89–3.02)	0.11

Notes: Others = not described, Mix = multiple radiotherapy techniques including 2DRT, 2DRT = two-dimensional general radiotherapy, 3DCRT = three-dimensional conformal.

**Table 5:** 5-year OS rate subgroup analysis.

	Number of studies	Heterogeneity	OR 95%CI	P
The combination mode of postoperative chemotherapy and radiotherapy				
Sequential chemoradiotherapy	10	*P* = .15, I^2^ = 32%	1.39 (1.21–1.59)	<0.00001
Concurrent chemoradiotherapy	5	*P* = .26, I^2^ = 25%	1.20 (0.84–1.71)	0.33
Others	3	*P* = .41, I^2^ = 0%	1.07 (0.83–1.71)	0.60
Radiotherapy technology				
3DCRT or (and) IMRT	11	*P* = .05, I^2^ = 46%	1.31 (1.04–1.66)	0.02
Mix	4	*P* = .95, I^2^ = 0%	1.48 (0.98–2.21)	0.06
Others	3	*P* = .41, I^2^ = 0%	1.07 (0.83–1.39)	0.59

Notes: Others = not described, Mix = multiple radiotherapy techniques including 2DRT, 2DRT = two-dimensional general radiotherapy, 3DCRT = three-dimensional conformal radiotherapy, IMRT = intensity modulated radiotherapy.

### 3.7. Sensitivity analysis and publication bias

**Figure 8.**
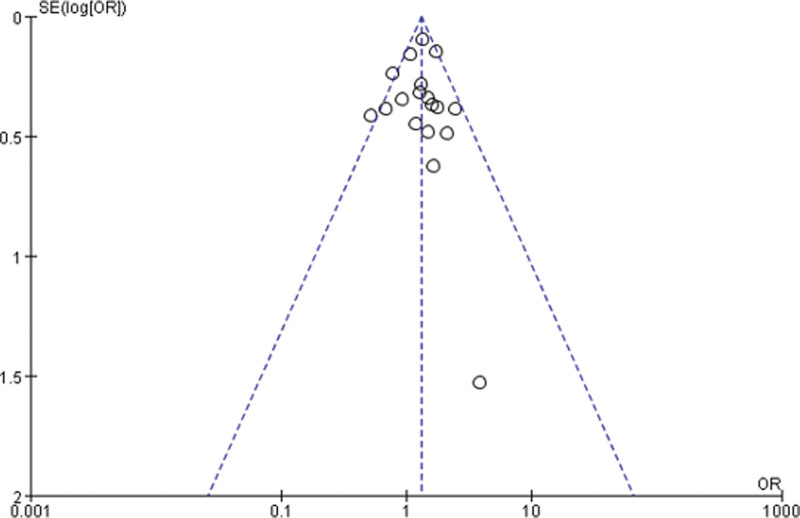
Funnel plot of 5-year OS rate.

Sensitivity analysis was performed for each meta-analysis, and 1 study was deleted at a time to assess the stability of the results. These analyses show that the corresponding OR values do not change obviously, indicating that our results are stable. Finally, the funnel plot was used to judge the bias degree of literature publication, and the funnel plot does not show any obvious evidence of asymmetry, suggesting that the possibility of publication bias is low (Fig. 8).

## 4. Discussion

Patients with N2 NSCLC have a high risk of local recurrence and distant metastasis after R0 resection. Adjuvant POCT can eliminate distant micrometastases and prolong DFS, which is considered as the standard treatment after R0 resection of stage N2 NSCLC.^[38]^ Although adjuvant POCT can bring survival benefits to patients, the effect is still not ideal, prompting researchers to explore the role of postoperative radiotherapy in postoperative adjuvant therapy for NSCLC. On the one hand, postoperative radiotherapy can kill the residual tumor cells and subclinical lesions in the surgical field, and reduce the local recurrence rate; on the other hand, it may lead to radiation injury and reduce the survival rate and quality of life of patients.^[39]^ In 1998, British scholars conducted meta-analysis of 9 high-quality randomized controlled trials (RCTs). The results showed that the 2-year OS rate and 2-year DFS rate in the postoperative radiotherapy group were 7% and 4% lower than those in the simple operation group. Stratified analysis showed that postoperative radiotherapy could not improve the survival rate of stage Ⅰ and stage Ⅱ patients, while stage III-N2 patients needed further clinical study.^[12]^ It is worth noting that in this meta-analysis, the vast majority of RCTs are treated with 2-dimensional general radiotherapy (2DRT), which has poor protection for normal tissue, resulting in severe radiation-related adverse reactions, which will offset the possible survival benefits of radiotherapy. With the advent of the era of precision radiotherapy, precision radiotherapy techniques represented by 3DCRT and IMRT have been widely used in the treatment of lung cancer, further reducing the nontumor death caused by radiation injury. Therefore, the clinical value of postoperative radiotherapy is again concerned.

In order to explore whether adjuvant POCT combined with postoperative radiotherapy can further improve the survival rate, reduce the risk of recurrence and metastasis, and make patients gain greater survival benefits. So for this problem, we have carried out a comprehensive system analysis. Our meta-analysis included 22 clinical studies involving 8959 patients with stage III-N2 NSCLC and systematic analyses of OS, DFS, LRFS, and DMFS rate. The results suggest that compared with POCT, POCRT can not only significantly increase the OS rate in patients with stage III-N2 NSCLC, especially in patients with N2 single-station lymph node metastasis, but also increase the DFS rate and reduce local recurrence, but it has no obvious advantage in improving the DMFS rate. The results of subgroup analysis showed that 3DCRT or IMRT could significantly increase the 3-year and 5-year OS rate. In addition, our results also show that the survival benefit of sequential chemoradiotherapy was significantly superior to that of concurrent chemoradiotherapy. We analyze the reasons for the following 2 points: (1) Sequential radiotherapy after chemotherapy makes patients more tolerant and easier to complete the entire treatment regimen; (2) patients can receive chemotherapy during the appointment waiting for radiotherapy, which is beneficial for more patients to get treatment in time. Of course, this result needs to be verified by more high-quality clinical studies.

Compared with the previous published similar studies,^[40]^ our study has expanded the scope of literature and added a lot of related studies published from 2016 to 2021. Therefore, the sample size of our study is larger and the population involved is wider. In addition, our study also conducted a subgroup analysis to screen out the population most likely to benefit from adjuvant POCRT and the best treatment model. Inevitably, our research also has its limitations: (1) Although the included studies have undergone strict quality evaluation and screening, there are few RCTs, and most of the included studies were retrospective studies, which may have selection bias and recall bias, thus affecting the level of evidence. (2) Incomplete consistency of chemotherapy cycles, chemotherapy regimens and radiation doses included in the study led to potential heterogeneity. (3) the available data on adverse reactions to radiotherapy are limited, so there is no systematic evaluation. (4) potential language bias and publication bias are inevitable.

In addition, immunotherapy represented by immune checkpoint inhibitors has achieved good results in the treatment of advanced NSCLC, and has become another important treatment method after surgery, chemotherapy, radiotherapy and targeted therapy. However, immune checkpoint inhibitors are mainly used in advanced patients with negative gene mutations, and there are few clinical studies on patients with stage III-N2 after R0 resection. Notably, after surgery and chemotherapy in patients (PD-L1 ≥ 1%) with stage II-IIIA NSCLC, atezolizumab was the only effective adjuvant immunotherapy agent, reducing the risk of disease recurrence or death by 34% compared with best supportive therapy.^[41]^ In the field of stage III unresectable NSCLC, the results of the PACIFIC study^[42]^ showed that consolidation therapy with durvalumab significantly extended survival in patients with no disease progression following concurrent chemoradiotherapy. Based on this result, the PACIFIC model has become the new treatment standard for such unresectable stage III NSCLC patients, and has been recommended by several clinical practice guidelines such as NCCN and CSCO. Therefore, it is worth looking forward to the application of immunocheckpoint inhibitors in patients with stage III-N2 after R0 resection.

In conclusion, Compared with POCT alone, adjuvant POCRT can significantly improve the overall survival rate of patients with NSCLC after R0 resection of stage III-N2, especially in patients with N2 single-station lymph node metastasis. Accurate radiotherapy techniques such as 3DCRT or IMRT are recommended, and postoperative sequential chemoradiotherapy is the best treatment mode.

## Author contributions

Data curation: Yaqi Pang, Lu Xu, Wanqiang Li, Dailong Li. Writing – original draft: Dailong Li, Wanqiang Li, Yaqi Pang. Writing – review & editing: Xinhua Xu.
